# EXPLAINABLE MACHINE-LEARNING FOR PREDICTING PREOPERATIVE FRAILTY PHENOTYPE USING ELECTRONIC HEALTH RECORDS

**DOI:** 10.1093/geroni/igac059.2127

**Published:** 2022-12-20

**Authors:** Mamoun Mardini, Catherine Price, Patrick Tighe, Todd Manini

**Affiliations:** University of Florida, Gainesville, Florida, United States; University of Florida, Gainesville, Florida, United States; University of Florida, Gainesville, Florida, United States; University of Florida, Gainesville, Florida, United States

## Abstract

Pre-operative frailty among patients is strongly associated with poor post-operative outcomes. Operationalizing frailty in clinical practice is challenging due to the lack of resources and pragmatic complexities. Feasible tools are needed to cover the scarcity in this area. Harnessing electronic health records (EHR) to screen pre-operative frailty and its related post-operative outcomes would be important for decision making and planning care management. This study aimed to validate an EHR-based machine learning model for pre-operative frailty ascertainment. Measures of the frailty phenotype (slowness, weight loss, exhaustion, low physical activity, and grip strength) were collected on approximately 14,000 patients (aged 65-100 years) by nurses housed in the UF Health pre-surgical center. Patients with at least 3 out of 5 syndromic components were considered frail. We utilized an explainable machine learning algorithm, eXtreme Gradient Boosting (XGBoost), to build our models to predict pre-operative frailty phenotype. We extracted the important predictors that contributed to predicting the outcome and evaluated their relationship with the outcome. The machine learning model achieved an area under the curve (AUC) of 0.71 in recognizing pre-operative frailty across all surgical specialties. The top five predictors for frailty phenotype were hemoglobin level, sex, education level, history of COPD, and diabetes. Using explainable machine learning approaches on EHR data provides a moderate mapping of the frailty phenotype in pre-operative settings. Funding: UF Claude D. Pepper Older Americans Independence Center P30AG028740 and R01 AG055337.

